# Self Examinations for Medical Students

**Published:** 1891-12

**Authors:** 


SELF EXAMINATIONS FOR MEDICAL STUDENTS.—3000 Questions on Medical Subjects. Arranged for Self Examination. P. Blakiston Son & Co., Philadelphia. 1891.

A compend of examinations in all the branches of medicine, in which the questions are given, with figures for reference to the standard text books in common use in each branch.

Being in concise form and so arranged that reference to the subjects examined on is made very easy, it commends itself to the student, and will be found of incalculable benefit in preparing himself thoroughly for the green room. R. W. W.

